# Multilocus Detection of Wolf *x* Dog Hybridization in Italy, and Guidelines for Marker Selection

**DOI:** 10.1371/journal.pone.0086409

**Published:** 2014-01-22

**Authors:** Ettore Randi, Pavel Hulva, Elena Fabbri, Marco Galaverni, Ana Galov, Josip Kusak, Daniele Bigi, Barbora Černá Bolfíková, Milena Smetanová, Romolo Caniglia

**Affiliations:** 1 Laboratorio di Genetica, Istituto Superiore per la Protezione e Ricerca Ambientale, Ozzano Emilia, Bologna, Italy; 2 Department 18/Section of Environmental Engineering, Aalborg University, Aalborg, Denmark; 3 Department of Zoology, Charles University in Prague, Prague, Czech Republic; 4 Life Science Research Centre, University of Ostrava, Ostrava, Czech Republic; 5 Department of Biology, Faculty of Science, University of Zagreb, Zagreb, Croatia; 6 Department of Biology, Faculty of Veterinary Medicine, University of Zagreb, Zagreb, Croatia; 7 Department of Agricultural and Food Science and Technology, University of Bologna, Bologna, Italy; 8 Faculty of Tropical AgriSciences, Czech University of Life Sciences, Prague, Czech Republic; Durham University, United Kingdom

## Abstract

Hybridization and introgression can impact the evolution of natural populations. Several wild canid species hybridize in nature, sometimes originating new *taxa*. However, hybridization with free-ranging dogs is threatening the genetic integrity of grey wolf populations (*Canis lupus*), or even the survival of endangered species (e.g., the Ethiopian wolf *C. simensis*). Efficient molecular tools to assess hybridization rates are essential in wolf conservation strategies. We evaluated the power of biparental and uniparental markers (39 autosomal and 4 Y-linked microsatellites, a melanistic deletion at the *β-defensin CBD103* gene, the hypervariable domain of the mtDNA control-region) to identify the multilocus admixture patterns in wolf *x* dog hybrids. We used empirical data from 2 hybrid groups with different histories: 30 presumptive natural hybrids from Italy and 73 Czechoslovakian wolfdogs of known hybrid origin, as well as simulated data. We assessed the efficiency of various marker combinations and reference samples in admixture analyses using 69 dogs of different breeds and 99 wolves from Italy, Balkans and Carpathian Mountains. Results confirmed the occurrence of hybrids in Italy, some of them showing anomalous phenotypic traits and exogenous mtDNA or Y-chromosome introgression. Hybridization was mostly attributable to village dogs and not strictly patrilineal. The melanistic *β-defensin* deletion was found only in Italian dogs and in putative hybrids. The 24 most divergent microsatellites (largest wolf-dog F_ST_ values) were equally or more informative than the entire panel of 39 loci. A smaller panel of 12 microsatellites increased risks to identify false admixed individuals. The frequency of F1 and F2 was lower than backcrosses or introgressed individuals, suggesting hybridization already occurred some generations in the past, during early phases of wolf expansion from their historical core areas. Empirical and simulated data indicated the identification of the past generation backcrosses is always uncertain, and a larger number of ancestry-informative markers is needed.

## Introduction

The routine application of multilocus genetic and genomic markers is providing deeper evidences on the evolutionary consequences of genetic admixtures. Hybridization in nature is no longer viewed as a sporadic, un-influential or merely negative process contrasting cladogenesis [1]. Natural hybrid zones are hot-spots of genetic diversity, where novel gene assemblages are filtered by natural selection, exposing genetic variability to the adaptive processes and eventually leading to hybrid speciation [2], [3]. Episodic hybridization events, although rare, may introduce genetic variation into isolated populations, contrasting the possible deleterious consequences of small effective size and inbreeding [4], [5]. In contrast, anthropogenic hybridization is usually viewed as a risk factor in conservation biology [6], [7]. Though genome integrity is not necessarily disrupted by hybridization [8], the long-term evolutionary consequences of introgression remain largely unpredictable. Introgression of alien genes may swamp genetic diversity [9], disrupt species-specific epistatic equilibria and local adaptations [10], and drive local populations [11] or entire species [12] to the verge of genetic extinction. However, recent findings indicated that introgression of genes from domestic species may also have unexpected beneficial consequences on the fitness of wild-populations [13]–15.

Wolf-like canids (genus *Canis*) evolved recently, during the last 2–4 million years [16], and retained the potential to hybridize in nature, originating new *taxa* that could rapidly adapt to prey community, landscape and climate changes [17]–[21]. In other cases, hybridization has deleterious consequences. Free-ranging or feral dogs (*C. lupus familiaris*) are widely distributed and can hybridize with wild canids [22]–[24]. Hybridizing dogs are threatening the survival of endangered species such as the Ethiopian wolf (*C. simensis*) [25], [26] and the genetic integrity of several populations of grey wolves (*C. lupus*) [27]–[32]. Most of the wolf populations in central and western Europe dramatically declined during the last few centuries. Legal protection and the expansion of wild ungulates led to spectacular wolf re-expansion waves [33], but their gene pools now risk to be polluted by hybridization with overwhelming numbers of free-ranging dogs [32]. The occurrence of wolf *x* dog hybridization has been documented by morphological observations and molecular identifications in the Iberian and Italian peninsulas, Scandinavia, Baltic countries and several areas of eastern Europe [27], [29], [34]. Consequently, conservation strategies require the assessment of wolf *x* dog hybridization rates and the development of cost-effective monitoring tools [35].

Unusual phenotypic traits may indicate hybridization, but their genetic determinants are often unknown. Moreover, introgressed variants may be undistinguishable from intraspecific variation [28], [36], [37]. Deep and ancient introgression is, in principle, better detected by molecular identifications [38]. Hybridization in wolves has been mainly analyzed by limited numbers of unlinked and presumably neutral autosomal markers (usually less than 30 microsatellites – STRs; [29], [32]). Uniparental markers (the hypervariable domain of the mtDNA control region - mtDNA CR1; Y-linked STR haplotypes [28], [39]–[42]) and recombination analyses of linkage groups have also been used [43]. However, few opportunistically selected markers have limited power to identify hybrids when the genetic divergence among the parental populations is lower than F_ST_ = 0.10–0.15. In these conditions, the identification of backcrosses behind the first 2 or 3 generations is tricky also with *c*. 40 STR or 100 single nucleotide polymorphism (SNP) markers [44]. Last-generation genomic tools promise to solve the problem [3], [15], [45], but genomic assays are expensive and still not widely used in conservation surveys of non-model species [46]. Expanding the number of markers is theoretically easy, but is offset by laboratory costs and increasing risks of introducing PCR or scoring errors in multilocus genotypes [47]. Therefore, selecting a minimum number of efficient markers is still the most productive strategy in applied conservation genetics [48], .

We planned this study to evaluate the power of biparental and uniparental molecular markers to identify presumptive wolf *x* dog hybrids sampled in Italy, which were previously identified by anomalous phenotypic traits or preliminary genetic analyses [28], [43], [50]. We compared the results of admixture analyses performed with 12, 24 and 39 autosomal STRs that were used in wolf hybridization studies in Europe [28]–[30], [42], [51]. Most of the published reports suggested a strict female wolf *x* male dog mating asymmetry, but the presence of dog mtDNA haplotypes was described in hybrid individuals in the Vancouver Island [52] and Latvia [30]. We assessed the directionality of hybridization by genotyping the mtDNA CR1, which contains diagnostic mutations for the identification of the unique Italian wolf haplotype W14 [40] and Y-STR haplotypes, which have different frequencies in wolves and dogs [39], [41]. Moreover, we assessed the presence of a functional melanistic deletion at the *β-defensin CBD103* gene (the *K*-locus), which determines black or darker-than-usual coat colours and could have been introduced into wolf populations via hybridization with dogs [13], [28]. Free-ranging dogs have various origins (village, hunting or shepherd dogs; random- or mixed-bred dogs), but differ from wild-living feral dogs and are usually not structured in stable populations. Thus, it is not easy to identify the parental sources of hybrids. Moreover, the composition of the reference parental samples may affect the results of admixture analyses [53]. Therefore, we used a reference panel of samples from different dog breeds and wolf populations that have chances to admix with wolves in Italy.

## Materials and Methods

### Sampling

We genotyped 271 wolves, dogs and putative hybrids, collected from 1996 to 2011 in Italy, Croatia, Czech and Slovak republics. We collected wolf samples from 3 populations: 1) Italy (WIT; *n* = 63; collected from the entire wolf range in the Apennines [54]); 2) Czech and Slovak republics (WCZ; *n* = 10; from the western Carpathians) and 3) Croatia (WHR; *n* = 26, from Dalmatia, Gorski kotar and Lika regions). All wolves had the typical wolf coat colour pattern and no apparent signal of morphological or genetic hybridization [28]. We collected samples from 3 dog groups: 1) village dogs in Italy (DIT; *n* = 31), sampled from the north and central Apennines and not selected based on their coat colours; 2) an undescribed local dog breed, “Lupino del Gigante”, bred in the northern Apennines and phenotypically similar to shepherd dogs, with variable grey, red, black, white and blue merle coats (named “Apennine dogs” in this study; DAP; *n* = 26); and 3) certified German Shepherd dogs bred in the Czech Republic (DCZ; *n* = 12). Samples of known or presumed hybrid origin were collected from 2 groups: 1) Czechoslovakian wolfdogs (WDCZ; *n* = 73), a hybrid breed of German Shepherd dogs *x* Carpathian wolf founders; 2) putative wild-living wolf *x* dog hybrids collected in Italy (HYIT, *n* = 30) and identified by their anomalous phenotypic traits (dog-like body shape, coat colour variations, presence of hind-leg spurs or white nails) or previous STR analyses [28], [43], [50].

We obtained the tissue samples from found-dead wolves legally collected by officers on behalf of the Italian Institute for Environmental Protection and Research (ISPRA), the Czech Agency of Nature Conservation and Landscape Protection, the Biology Department, Faculty of Veterinary Medicine, Zagreb University, Croatia. We obtained additional samples from legally hunted wolves in Croatia, according to quotas defined by the Croatian Commission for monitoring large carnivore populations and approved by the Croatian Ministry for Environmental and Nature Protection. No animal was sacrificed for the purposes of this study. Blood and saliva samples from dogs and Czechoslovakian wolfdogs were collected by veterinaries that, according to Act 246/1992, sampled only animals in healthy conditions with permission and assistance of the owners and with all the possible efforts to minimise stress. We stored tissue and blood samples at −20°C in 10 volumes of 95% ethanol, or in 2 volumes of a Tris/SDS buffer, respectively. Saliva samples were dry-stored. We extracted DNA using a QIAGEN DNeasy tissue extraction kit (Qiagen Inc, Hilden, Germany) in a robotic liquid handling system MULTIPROBE II^EX^ (Perkin-Elmer). In this study, we reanalyzed all the samples.

### Selection of Molecular Markers

We selected a panel of 39 canine autosomal STRs that were used in some of the most recent studies on wolf population genetics and hybridization in Europe [28], [29], [39], [42], [43], [50], [51], [55]–[60], which includes: 1) 12 STRs used in a 10-year long non-invasive wolf monitoring project in Italy [61]; 2) 12 STRs used in a hybridization study by Godinho et al. [29]; and 3) 15 STRs from the Finnzymes Canine multiplex kit (Finnzymes, Thermo Scientific Canine Genotypes™); one of them, the *Amelogenin* marker, was used to sex the individuals (the other 4 of the 19 STRs included in this kit were not used because they showed confusing electropherograms). These 39 STRs were amplified in 4 PCR multiplexes using the Qiagen Multiplex PCR Kit (Qiagen, GmbH-Hilden, Germany). Paternal haplotypes were identified using 4 Y-STRs (MS34A, MS34B, MSY41A and MS41B; [41]) that were amplified in a multiplex reaction. The hypervariable part of the mtDNA CR1 (350 bp) was amplified and sequenced following Randi et al. [40]. A dominant 3-bp deletion (named *K^B^* or *CBD103^ΔG23^*) at the *β-defensin CBD103* gene (the *K*-locus [36]) was genotyped following Caniglia et al. [28]. Amplifications were carried out in 10–20 µl reactions, using 1–2 µl DNA solution (containing *c.* 20–40 ng of DNA). Negative (no DNA in PCR) and positive (samples with known genotypes) controls were used to detect laboratory contaminations. All samples were independently replicated twice to assess the occurrence of allelic dropout and false alleles. Details on the selected markers, primers and PCR profiles are reported in Text S1 and in Table S1.

The amplicons were analysed in an ABI DNA sequencer 3130XL (Applied Biosystems; Foster City, CA), using the software Genemapper 4.0 for STRs and SeqScape 2.5 for sequences. The mtDNA sequences were aligned using Clustal W [62] in BioEdit [63]. Identical haplotypes were collapsed using DnaSP 5 [64] and blasted in GenBank. Allele binning and check for null STR alleles were performed in Microchecker [65] with an adjusted *P* value corresponding to *α* = 0.05 after Bonferroni correction [66]. The power of the STRs to identify each unique genotype was evaluated calculating the probability-of-identity values (PID and PIDsibs; [67]) in GenAlEx 6.41 [68].

### Estimates of Genetic Variability and Population Structure

The multilocus genotypes determined at 39 STRs in the complete data set (*n* = 271; 8 sampled groups: DIT, DAP, DCZ, WIT, WHR, WCZ, WDCZ and HYIT) were analyzed in GenAlEx to estimate: 1) allele frequency by locus and population, observed (H_O_) and unbiased expected (UH_E_) heterozygosity, mean number of alleles per locus (N_a_) and the number of private alleles per population (N_p_); 2) AMOVA (analysis of molecular variance [69]) and Weir and Cockerham’s average and pair-wise F_ST_ values [70]; 3) the frequency distributions of mtDNA CR1 and Y-STR haplotypes, and melanistic *K* ^B^ deletion. Genetix 4.05 [71] was used to compute the fixation index F_IS_ and to test for departures from Hardy-Weinberg and linkage equilibrium (HWLE) for each locus and population. A subset of the 24 most discriminating STRs was identified based on F_ST_ distances between wolves and dogs, and confirmed in Whichloci analyses [72], performed using the “allele frequency differential” and the “whichrun assignment” methods [73], [74]. A third marker subset included the 12 STRs used in the monitoring project of the Italian wolf population [75], [76].

Clustering and assignment testing were performed by: 1) a discriminant analysis of principal components computed by the Adegenet package (DAPC [77] in R; www.r-project.org), which maximizes the among-group divergence while minimizes the within-group variance, thus improving the discrimination of populations poorly differentiated as compared to standard principal component methods; 2) the Rannala and Mountain’s [78] assignment method in GeneClass 2.0 [79]; 3) the Bayesian clustering model (assuming HWLE in the genetic clusters) implemented in Structure 2.3 [80]; 4) a non-Bayesian clustering procedure (that does not assume HWLE in the clusters) implemented in Flock 2.0 [81]. First, we used Structure to infer the optimal partition of the 8 sampled groups, assuming *K* from 1 to 12, with 2 independent runs for each *K* with 400 000 MCMC and discarding the first 40 000 burn-ins, using the “*admixture*” and independent allele frequency “*I*” models, and no prior information (option “*usepopinfo*” not activated). The Δ*K* statistics was used to identify the highest rate of increase in the posterior probability Ln*P*(D) of the data between each consecutive *K* [82]. Based on the first Structure results, admixture analyses were performed again assuming 4 reference groups (DIT, DAP, DCZ and WIT) for the assignment of the putative Italian wolf *x* dog hybrids (HYIT), using 39, 24 and 12 STRs. Structure was run with *K* from 1 to 8, with 400 000 MCMC and 40 000 burn-ins, with the option “*usepopinfo*” activated or not. In the former case, we assumed that reference wolves and dogs were *a priori* correctly identified and assigned to their own clusters (*popflag* = 1), while the putative hybrids were left to be assigned (*popflag* = 0). The estimated allele frequencies of the wolf and dog reference clusters were not affected by the allele frequencies of the other samples (option *updatepfrompopflagonly* activated).

The software Flock implements a non-Bayesian clustering algorithm based on reiterated allocations that promises efficient partitioning of the admixed samples in groups of homogeneous genotypes, also if putative parental populations are not sampled, independent of any genetic model (i.e., HWLE is not assumed). Flock was used to partition samples DIT, DAP, DCZ, WIT and HYIT, with reference groups varying from 1 to 8, initial random choice of samples, 50 runs and 20 re-allocations per run (LOD threshold for allocation to reference groups = 0). Admixture inference may be difficult when model assumptions are not met and if small numbers of markers are used (but also if the number of loci is large; [83]). For instance, when an unknown number of *K* parental populations must be inferred simultaneously to the admixture coefficients, both overfitting (too large *K* values) and false admixtures may results, particularly if the sampled populations diverged moderately (F_ST_ <0.10; [84]). Hence, false positives (error type I), namely individuals with false admixed ancestry, might arose by chance. In this study, we explored the risk of false admixtures using Baps [84], which produces null distributions for the admixture expected by chance that are used to identify significant admixtures at a given *p*-value [85].

The power of the 39, 24 and 12 STRs to correctly detect *a priori* known parentals, hybrids and backcrosses was determined by simulations using HybridLab [86]. We randomly selected 60 reference wolves and 60 reference dogs among WIT and DIT to generate 60 simulated genotypes in each of the following classes: first (F1) and second (F2) generation hybrids, first (BC1W, BC1D), second (BC2W, BC2D) and third (BC3W, BC3D) generation backcrosses with wolves and dogs, respectively. The simulated genotypes were then analyzed in Structure with the “*admixture*” and the “*I*” models, without prior population information. The proportion of individuals correctly assigned to each class led to define the appropriate threshold value to use in the admixture analyses.

The software NewHybrids 1.1 [87] was used to compute the posterior probability that each genotype belongs to each of the following 6 classes: wolf (W) and dog (D) parentals, F1 and F2, backcrosses of F1 with dogs (BC1D) and with wolves (BC1W). Posterior distributions were evaluated after 10^5^ iterations of the Monte Carlo Markov chains, following a burn-in period of 10^4^ iterations, without using any individual or allele frequency prior information, with “Jeffreys-like” or “Uniform” priors for mixing both proportions and allele frequencies.

## Results

### Genetic Variability and Wolf-dog Divergence

The 39 STRs were polymorphic, except CPH5 and PEZ5 (monomorphic in DCZ), showing from 5 (at locus FH2096) to 23 (at locus FH2137) alleles per locus. The number of alleles and private alleles was higher in dogs than in wolves (Table 1). Heterozygosity varied from Ho = 0.46 - UHe = 0.48 in WIT to Ho = 0.69 - UHe = 0.71 in DIT. DIT, WIT, HYIT and WHR were not in HWE, showing significantly positive F_IS_ values. We found no null and false alleles, and no occurrence of allelic dropout. Values of PID and PIDsibs were very low, and all genotypes were unique (Table 1). The proportions of significant pairwise correlations among loci were low (from 0.7% in DCZ to 3.0% in WCZ), indicating no departures from LE. We found a total of 17 Y-STR haplotypes (Table 2). WHR and DIT were the most variable groups. HYIT showed 2 haplotypes (YH17 and YH26) shared with WIT, plus haplotype YH5, shared with dogs and WDCZ, and the private haplotype YH32. There were 19 mtDNA CR1 haplotypes in total (Table 3). DIT had the highest number of haplotypes (8). All WIT had the diagnostic W14 haplotype, that was found in 26/30 (87%) of HYIT. HYIT showed also haplotypes D15 (1), shared with both DIT and DAP, and W16 (3) that was previously identified in Bulgarian wolves [40]. We detected the *K^B^* melanistic deletion only in samples from Italy, with similar *K^B^*/*K* ^+^ heterozygote frequencies in DIT (0.20), DAP (0.31) and HYIT (0.23).

**Table 1:** Genetic variability estimated at 39 autosomal microsatellite loci (STR) and at the *K^B^* melanistic deletion on the *β-defensin CBD103* gene in the wolf, dog and putative hybrid sampled groups used in this study.

Group*	*n* a	Na/Np^b^	Ho^c^	UHe^d^	F_IS_ e	%LE^f^	PID^f^	PIDsib^g^	*K* ^+^/K^+h^	*K^B^/K* ^+h^	*K^B^/K^B^* ^h^
DIT	31	7.1/35	0.69	0.71	0.07*	2.4	2.0E-39	9.1E-16	21 (0.70)	8 (0.20)	2 (0.07)
DAP	26	5.0/4	0.63	0.64	0.03	2.0	2.5E-31	1.8E-13	17 (0.65)	8 (0.31)	1 (0.04)
DCZ	12	3.3/0	0.50	0.48	−0.03	0.7	5.4E-20	1.8E-09	12 (1.00)	0	0
WIT	63	4.0/4	0.46	0.48	0.06*	1.3	1.1E-21	3.8E-10	63 (1.00)	0	0
WCZ	10	4.1/6	0.58	0.66	0.13*	3.0	7.8E-29	6.2E-13	10 (1.00)	0	0
WHR	26	5.6/18	0.68	0.70	0.03	1.3	4.2E-35	1.0E-14	26 (1.00)	0	0
WDCZ	73	4.7/12	0.54	0.54	0.00	1.6	8.2E-24	4.7E-11	73 (1.00)	0	0
HYIT	30	5.3/5	0.53	0.57	0.08*	1.9	2.6E-26	8.5E-12	23 (0.77)	7 (0.23)	0

* DIT = Village dogs from Italy; DAP = Apennine dogs; DCZ = German Shepherd dogs from Czech and Slovakian republics; WIT = Wolves from Italy; WCZ = Wolves from Carpathian Mountains; WHR = Wolves from Croatia; WDCZ = Czechoslovakian wolfdogs; HYIT = putative wolf *x* dog hybrids from Italy.

a *n* = sample size.

b Na = average number of alleles per STR locus; Np = total number of private STR alleles in each sampled group.

c Ho = average observed heterozygosity over 39 autosomal STRs.

d UHe = average expected heterozygosity (unbiased) over 39 autosomal STRs.

e F_IS_ = deviation from Hardy-Weinberg equilibrium (* *P*<0.01).

f %LE = proportion of significant correlations (*P* = 0.05, Bonferroni corrected) among 39×39 pairwise STR comparisons.

g PID and PIDsib = Hardy-Weinberg probability-of-identity among unrelated and full sib individuals in the sampled groups, computed using 39 autosomal STRs.

h number and frequency (in parenthesis) of genotypes at the *β-defensin CBD103* gene: *K* ^+^/K^+^ = homozygotes wild-type (no deletion); *K^B^/K* ^+^ = heterozygotes for the *K^B^* melanistic deletion; = *K^B^/K^B^* = homozygotes for the *K^B^* melanistic deletion.

**Table 2:** Distribution of the Y-linked microsatellite haplotypes in the wolf, dog and putative hybrid sampled groups.

Y haplotype	DIT	DAP	DCZ	WIT	WCZ	WHR	WDCZ	HYIT	S-2001^a^	I-2010^b^
YH01							19		J	H4
YH05	6	14	6				10	2	L	H3
YH06	6									
YH08						3			C	–
YH09						1			–	–
YH11					2	3			G	–
YH16					1	1			K	–
YH17				27				13	–	H1
YH20						6			–	–
YH24	1								–	–
YH26				4				2	Q	H2
YH27	1								–	–
YH28	1								–	–
YH31					2	1			–	–
YH32								1	–	–
YH33						1			I	–
YH34	3	1							–	
Total males	18	15	6	31	5	16	29	18		
Total haplotypes	6	2	1	2	3	7	2	4		
Private haplotypes	4	0	0	0	0	4	1	1		

a S-2001 = haplotype identifications as named in Sundqvist et al. [41].

b I-2010 = haplotype identifications as named in Iacolina et al. [39].

**Table 3:** Distribution of the mtDNA CR1 haplotypes in the wolf, dog and putative hybrid sampled groups.

CR*	DIT	DAP	DCZ	WIT	WCZ	WHR	WDCZ	HYIT
D01^a^	6	3						
D05^a^	1	2						
D06^a^			1					
D08^a^	3							
D09^a^	3	5						
D10^a^	2							
D13^a^			2				47	
D14^a^	13	3	3					
D15^a^	2	13						1
D16^a^	1							
D17^a^			1					
D18^a^			4				22	
H6^b^					2			
H14^b^					6			
W1^c^						13		
W2^c^						1		
W6^c^						12		
W14^a^				63				26
W16^a^								3
Total samples	31	26	11	63	8	26	69	30
Total haplotypes	8	5	5	1	2	3	2	3
Private haplotypes	3	0	2	0	2	3	0	1

* mtDNA CR1 haplotypes identifications as named in Randi et al. [40] ^a^, Pilot et al. [90] ^b^ and Gomeri et al. 98 ^c^.

**Figure 1:**
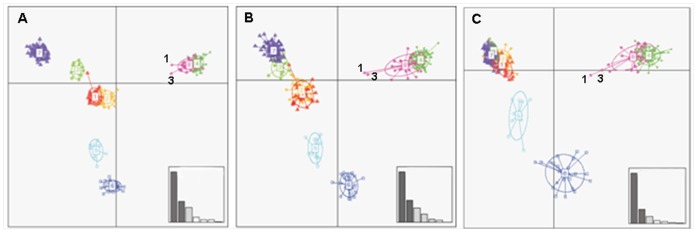
Discriminant analysis of principal components (DAPC) of wolf, dog and wolf *x* dog hybrids genotyped with 39 (A), 24 (B) and 12 (C) autosomal microsatellites. Sampling groups: 1) village dogs sampled in Italy (DIT; *n* = 31); 2) “Lupino del Gigante” dogs from Italy (DAP; *n* = 26); 3) German Shepherd dogs from Czech Republic (DCZ; *n* = 12;); 4) wolves in Italy (WIT; *n* = 63); 5) wolves in Czech and Slovak republics (WCZ; *n* = 10); 6)wolves in Croatia (WHR; *n* = 26); 7) certified Czechoslovakian wolfdogs (WDCZ; *n* = 73); and 8) putative wolf *x* dog hybrids (HYIT; *n* = 30) collected in Italy and identified by their anomalous phenotypic traits (dog-like body shape, coat colour variations, presence of hind-leg spurs or white nails), or by previous microsatellite analyses. Black numbers indicate the most probable F1 (sample n. 1) and F2 (sample n. 3) individuals as determined by Structure and NewHybrids analyses. The first principal component PC I (abscissa) explains 51.48%, 49.96% and 63.65% of the total genetic variance shown by genotypes determined at 39, 24 and 12 microsatellites, respectively. The corresponding second principal component PC II (ordinate) explains 21.25%, 21.93% and 18.19% of the total genetic variance. The inserts (low right corners) indicate the proportion of genetic variability explained by the first 6 eigenvalues.

Genetic diversity at autosomal and uniparental markers was significantly (*P*<0.001) partitioned among the 8 groups, with F_ST(phiPT)_ = 0.25 (39 STRs), 0.52 (Y-STRs) and 0.49 (mtDNA CR1). Pairwise F_ST_ varied deeply among groups (min F_ST_ = 0.01 between WIT and HYIT; max F_ST_ = 0.42 between WIT and DCZ), and among loci (min F_ST_ = 0.09 at locus FH2001; max F_ST_ = 0.45 at locus U253). The 24 wolf-dog most divergent STRs, identified by both single-locus F_ST_ and Whichloci selections, were: C20.253, CPH9, CPH4, RE247M23, CPH12, AHTh260, INU030, AHT103, CPH2, CPH14, AHTk253, C27.442, CPH5, FH2010, AHTk211, AHT132, C13.758, C09.173, AHT111, AHTh171, REN169D01, INU055, FH2848 and AHT137. Wolf-dog average F_ST_ computed using 24 STRs (0.31) was higher than with 39 STRs (0.25) or 12 STRs (0.25). A DAPC plot obtained using 39 STRs showed that all groups were sharply distinct except the partially overlapping Italian wolves and hybrids (Fig. 1). Multivariate distances among groups decreased progressively using 39, 24 or 12 STRs, but wolves and dogs were more distant with the most divergent 24 STRs. Two individuals, the most probable F1 and F2 in Structure and NewHybrids analyses (see Table 4), were roughly intermediate between Italian wolves and dogs (Fig. 1B).

**Table 4:** Identifications of the 30 putative wolf *x* dog hybrid samples used in this study.

						Structure *popinfo* = 0^g^			Structure *popinfo* = 1^h^			GeneClass i			Flock j	Baps k	NH^l^	
ID^a^	Y^b^	Ph^c^	CR^d^	Y-STR^e^	*K* f	39	24	12	39	24	12	39	24	12	39	39	39	ID^m^
1	1997	Dog-like	D15	YH17	+	0.439	0.472	0.405	0.448	0.468	0.382	99.9	100	98.4	HY	HY	F1	F1
2	1999	Spur	W14	YH17	+	0.690	0.700	0.914	0.684	0.706	0.866	100	100	98.7	HY	HY	F2-BC	F2
3	2007	unknown	W14	*female*	B	0.625	0.459	0.602	0.629	0.470	0.600	100	100	100	HY	HY	F2	F2
4	1999	Spur; dark coat	W14	YH17	B	0.886	0.934	0.960	0.860	0.890	0.862	99.9	99.8	52.7	HY	W	BC	BC
5	2001	White nails	W14	*female*	+	0.760	0.833	0.957	0.759	0.811	0.853	100	100	99.1	HY	HY	BC	BC
6	2006	White nails	W14	*female*	+	0.833	0.900	0.959	0.818	0.842	0.884	100	100	95.1	HY	HY	BC	BC
7	2011	Brown coat	W14	YH17	+	0.855	0.809	0.839	0.846	0.798	0.794	100	100	99.9	HY	HY	BC	BC
8	2007	Spur	W14	*female*	+	0.795	0.774	0.989	0.772	0.754	0.971	100	100	17.9	HY	HY	BC	BC
9	2007	Dark coat	W16	YH26	+	0.995	0.987	0.989	0.984	0.963	0.968	2.5	71.1	94.3	HY	W	W	BC
10	2006	wild-type	W14	YH17	+	0.972	0.946	0.979	0.919	0.886	0.920	99.9	99.9	99.8	HY	W	W	BC
11	2011	wild-type	W14	YH32	+	0.661	0.592	0.686	0.646	0.591	0.629	100	100	100	HY	HY	BC	BC
12	2012	wild-type	W14	*female*	+	0.882	0.971	0.993	0.871	0.930	0.982	100	97.0	23.1	HY	W	BC	BC
13	2012	unknown	W14	YH05	+	0.837	0.740	0.958	0.826	0.791	0.929	100	100	66.6	HY	HY	BC	BC
14	2009	unknown	W14	YH05	+	0.988	0.979	0.977	0.971	0.956	0.956	95.9	44.9	87.4	W	W	W	BC
15	2011	wild-type	W14	YH17	+	0.940	0.977	0.992	0.906	0.941	0.980	99.9	99.6	7.0	W	W	W-BC	BC
16	2006	unknown	W14	*female*	+	0.977	0.977	0.958	0.951	0.947	0.901	97.9	68.4	99.5	W	W	W	BC
17	2002	Black coat	W14	YH17	B	0.997	0.996	0.988	0.993	0.989	0.963	6.3	2.4	75.3	W	W	W	IG
18	2009	Black coat	W14	*female*	B	0.998	0.997	0.993	0.995	0.992	0.980	0.0	0.0	9.2	W	W	W	IG
19	2009	Dog-like; dark	W14	YH17	B	0.996	0.995	0.993	0.989	0.982	0.982	1.3	43.1	36.3	W	W	W	IG
20	2000	Black coat	W14	*female*	B	0.997	0.996	0.991	0.993	0.988	0.978	6.5	10.0	46.9	W	W	W	IG
21	2012	White nails	W14	YH17	+	0.998	0.997	0.994	0.994	0.991	0.985	0.0	8.2	4.3	W	W	W	IG
22	2011	unknown	W16	*female*	+	0.998	0.997	0.993	0.994	0.991	0.978	0.0	0.0	54.5	W	W	W	IG
23	2010	unknown	W16	YH26	+	0.995	0.993	0.986	0.979	0.964	0.935	98.6	99.0	99.0	W	W	W	IG
24	2006	unknown	W14	YH17	B	0.995	0.997	0.989	0.986	0.992	0.964	13.4	0.0	30.2	W	W	W	IG
25	2007	wild-type	W14	YH17	+	0.997	0.997	0.990	0.989	0.986	0.962	0.0	6.4	12.5	W	W	W	FP
26	2006	wild-type	W14	*female*	+	0.995	0.994	0.978	0.980	0.969	0.905	9.6	47.5	99.4	W	W	W	FP
27	2007	wild-type	W14	YH17	+	0.996	0.995	0.990	0.988	0.980	0.958	8.8	25.2	84.4	W	W	W	FP
28	1997	wild-type	W14	*female*	+	0.997	0.996	0.981	0.980	0.966	0.867	2.6	63.4	95.6	W	W	W	FP
29	2010	wild-type	W14	YH17	+	0.997	0.996	0.992	0.988	0.979	0.962	11.4	46.1	97.3	W	W	W	FP
30	2006	wild-type	W14	YH17	+	0.996	0.993	0.979	0.984	0.979	0.944	4.1	27.2	77.9	W	W	W	FP

a ID = sample identification number.

b Y = year of sampling.

c Oh = phenotype traits.

d CR = mtDNA control region haplotypes.

e Y-STR = Y-linked STR haplotypes detected in males.

f *K* = melanistic deletion at the *β-defensin CBD103* gene:+ = homozygote wild-type (no deletion), B = heterozygote for the *K^B^* melanistic deletion.

g and ^h^ Structure = individual proportion of assignment in Structure admixture analyses to the Italian wolf cluster with 39, 24 and 12 microsatellites, with option usepopinfo not activated (*popinfo* = 0) or activated (*popinfo* = 1).

i GeneClass = probability of assignment to a distinct cluster (admixed genotypes) with 39, 24 and 12 microsatellites performed in GeneClass.

j Flock = assignment obtained through the non-Bayesian clustering procedure implemented in Flock to an Italian wolf (W) or hybrid (HY) cluster.

k Baps = assignment to an Italian wolf (W) or an admixed (HY) cluster with 39 microsatellites as inferred using Baps.

l NH = assignment to parental Italian wolf (W), F1, F2 or first generation backcross (BC) genotypic classes obtained with NewHybrids.

m ID = final identification of each sample as a likely F1, F2, backcross (BC), introgressed (IG) or false admixed (FP) genotype, based on a qualitative consensus of all the probabilistic admixture analyses.

### Population Clustering and Admixture Analyses

**Figure 2:**
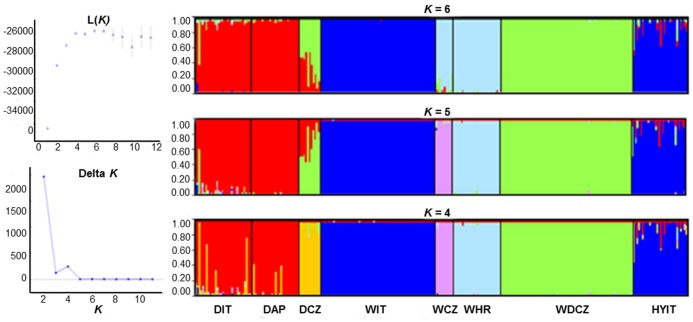
Structure analyses performed to infer the optimal partition of 8 sampled groups (A): DIT = village dogs in Italy; DAP = Apennine dogs; DCZ = German Shepherd; WIT = wolves in Italy; WCZ = wolves in Czech and Slovak republics; WHR = wolves in Croatia; WDCZ = Czechoslovakian wolfdogs; HYIT = putative wolf *x* dog hybrids collected in Italy; (genotyped at 39 autosomal microsatellites). The posterior probability Ln(*K*) of the data and the statistics Δ*K* were used to identify the optimal *K* = 4 (averages of 2 independent runs). Plots of individual assignment probability to each inferred cluster are shown (B) for optimal *K* = 4, 5 and 6. Structure was run assuming *K* from 1 to 12, with 400 000 MCMC and discarding the first 40 000 burn-ins, using the “*admixture*” and independent allele frequency “*I*” models, and no prior information (option “*usepopinfo*” not activated).

The probability of the data reached a plateau at Δ*K* = 4–6, with minimum Ln*P*(D) values at *K* = 6 in Structure analyses performed with 39 STRs and 8 groups (Fig. 2A). At *K* = 4 wolves and dogs were split into 4 clusters: dogs, WDCZ, WIT, WCZ plus WHR. At *K* = 5 and *K* = 6 the 3 wolf groups (WIT, WCZ and WHR) were assigned to 3 distinct clusters (Fig. 2B). WIT were not admixed while 14/30 (47%) of the putative hybrids showed signals of Italian wolf *x* dog admixture with *q*_i_ values ranging from 0.509 to 0.953. The main contributions to admixture derived from WIT, DIT and DAP (Table S2). There was no apparent contribution from the 2 non-Italian wolf populations, with the exception of one sample that also showed the private Y-haplotype YH32. Flock results with *K* = 7 and 8 were concordant: the 3 wolf groups, DWCZ and HYIT were correctly assigned to different groups while the 3 dog groups were not separated.

**Figure 3:**
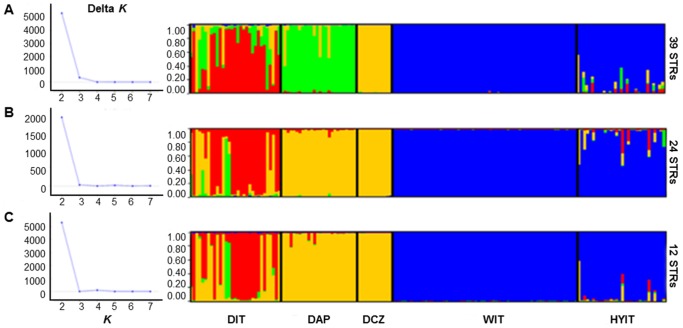
Structure analyses performed on the putative Italian wolf *x* dog hybrids (HYIT), assuming 4 reference groups (DIT, DAP, DCZ and WIT), at 39 (A), 24 (B) and 12 (C) microsatellites. Structure was run with *K* from 1 to 8 (left side: values of Δ*K*; Evanno et al. [82]), with 400 000 MCMC and 40 000 burn-ins, with option “*usepopinfo*” not activated.

We compared the efficiency of the 39, 24 and 12 STRs to assign HYIT to their most likely parental groups (DIT, DAP, DCZ and WIT). Δ*K* stabilized at *K* = 3–4 in Structure analyses (Fig. 3). All WIT were assigned to their own cluster with *q*_i_ >0.993, with the exception of one sample. In contrast, 13 (43%) to 15 (50%) of the 30 HYIT genotypes showed detectable signals of admixture with *q*_i_ values ranging from 0.405 to 0.988. The other 15 samples did not show signals of admixture at their STR genotypes. The main contributions to admixture derived from the Italian wolves (cluster 4 in Table S3). Structure run with *popflag* = 1 for wolves and dogs showed the same results (Table S3), while Flock did not split WIT from HYIT and did not detect admixtures (not shown).

**Figure 4:**
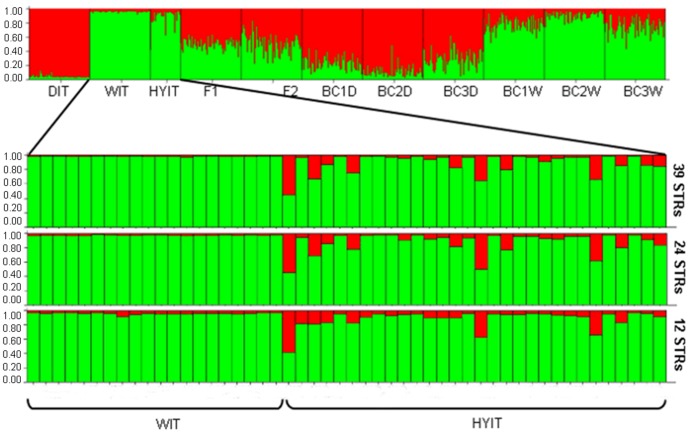
Structure analyses of empirical (DIT, WIT and HYIT) and HybridLab-simulated genotypes identified using 39 microsatellites. F1 and F2 between wolf and dogs; BC1 = first, and BC2 = second backcross with dogs (D) or wolves (W); BC3D and BC3W = F2 backcrossed with dogs or wolves, respectively. Structure was run with *K* = 2; *admixture* and *I* models, *popflag* = 0. Details of the individual proportion of admixture in the Italian wolves (WIT) and putative hybrid (HYIT), genotyped with 39 (top), 24 (mid) or 12 (bottom) microsatellites are showed.

**Figure 5:**
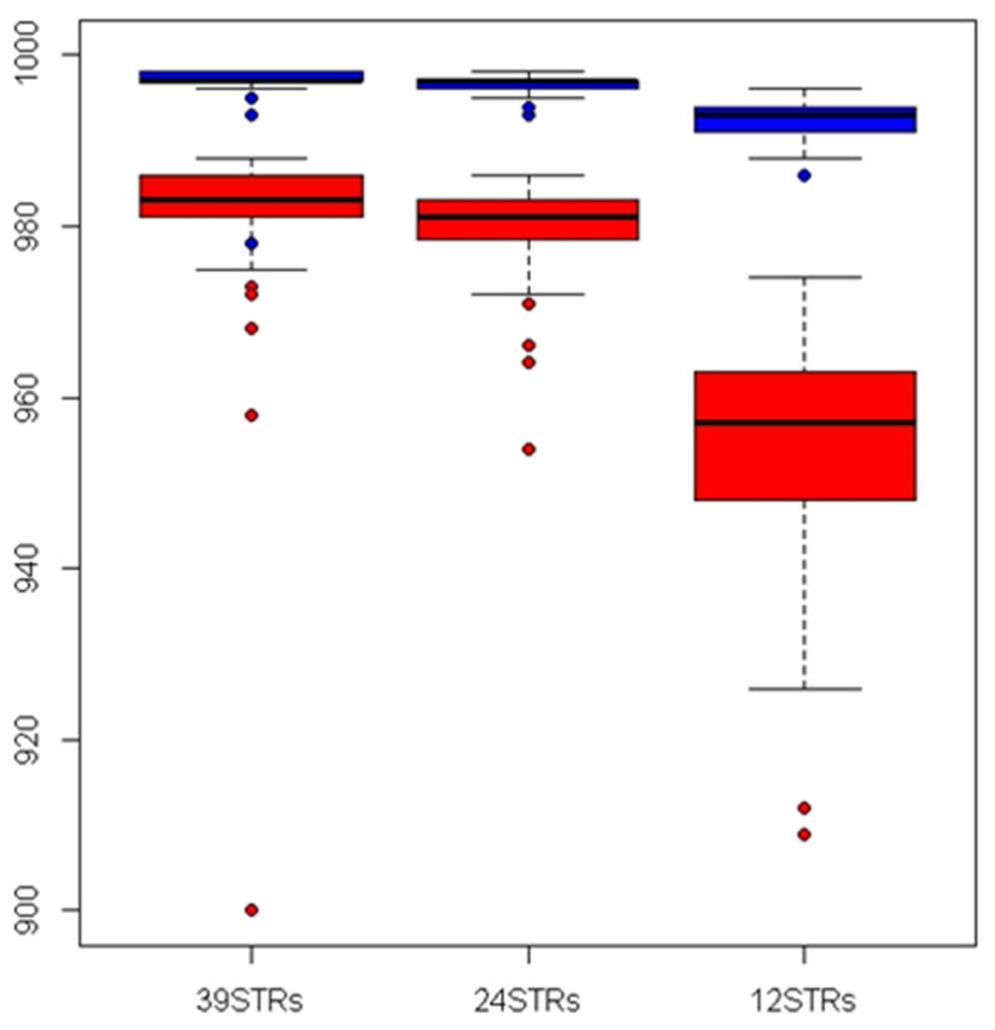
Average proportion of membership (*q*_i_, upper boxplots) of wolves from Italy (WIT) to the wolf cluster and lower boundary of their 90% credibility intervals (CI; lower boxplots), computed on genotypes at 39, 24 or 12 microsatellites.

The 5 genotypic classes simulated in HybridLab were correctly identified by Structure (*K* = 2; Fig. 4) with 39, 24 or 12 STRs. All simulated F1, F2 and BC1 were correctly assigned while *c*. 20% of the BC2 were confused with parental dogs or wolves. Decreasing the number of loci yielded decreasing values of the average proportion of membership in dogs (*Q*_i_ = 0.973, 0.968 and 0.958) and wolves (*Q*_i_ = 0.985, 0.980 and 0.960 with 39, 24 and 12 STRs respectively) due to increasing background noise in both wolves and dogs (Fig. 4). Consequently, the 90% confidence interval (CI) values broadened, thus increasing the uncertainty of the assignments, particularly when Structure was run with 12 STRs (Fig. 5). The risk of false positives (false admixed individuals) was inversely proportional to the number of STRs as indicated by BAPS results: admixture analyses based on 100 simulations for spurious admixture coefficients yielded 9 (30%), 8 (23%) and only 5 (17%) significantly admixed individuals (*P* = 0.05) with 39, 24 and 12 STRs, respectively.

### Hybridization and Multilocus Introgression

Structure results with *popinfo* = 0 and 39 STRs showed that, at the threshold *q*_i_ = 0.985, all the Italian wolves were assigned to the same cluster with one exception. Sixteen (53%) of the 30 HYIT were identified as admixed (Table 4). Structure results with 24 and 12 STRs (and *popinfo* = 0) yielded 15 (50%) and 10 (33%) admixed HYIT at thresholds *q*_i_ = 0.980 and 0.960, respectively. At the same thresholds, Structure results obtained with *popinfo* = 1 yielded 20 (67%), 23 (77%) and 21 (70%) admixed genotypes with 39, 24 and 12 STRs respectively. Structure assignments were consistent for the 16 admixed genotypes showing the lowest *q*_i_ values. These genotypes were also identified by GeneClass, with the exception of sample n. 9. Flock identified as admixed 13 of these genotypes with 39 STRs, which were, however, assigned to the WIT cluster if analysed with 24 or 12 STRs. Only 9 of these genotypes were identified as admixed by Baps (Table 4). NewHybrids with 39 STRs showed that: 1) all dogs had posterior probability *P*>0.999 to be “parentals”, except 4 samples; 2) all Italian wolves had posterior probability *P* = 1.000 to be “parentals”, with one exception (see Structure results above); 3) among HYIT there was one F1 (n. 1), 2 F2 (n. 2 and n. 3), and 9 backcrosses (Table 4). The other 18 samples (60%) were assigned to the parental wolf population.

Twelve of the 16 admixed genotypes also showed one or more anomalous phenotypic traits, the *K* ^B^ deletion, variant mtDNA or Y-STR haplotypes (Table 4). These 16 genotypes were finally identified as F1 (n. 1), F2 (n. 2 and 3) or backcrosses (BC; n. 4 to n. 16). Eight genotypes (n. 17 to 24) did not show any admixture signal at their STR genotypes but showed phenotypic anomalies, the *K* ^B^ allele or the mtDNA haplotype W16 and were finally identified as introgressed (Table 4). The remaining 6 samples (n. 25 to 30) were identified as presumptive hybrids only in Structure analyses with 12 STRs.

## Discussion

All genotyped wolf, dog and hybrid samples were variable at autosomal and uniparental markers. Italian wolves showed the lowest genetic diversity, probably as a consequence of long-lasting genetic isolation south of the Alps and a recent bottleneck [89], clustering separately from the other studied populations. Wolf populations in eastern Europe, though experienced less dramatic bottlenecks or had persistent gene flow with neighbouring populations [16], exhibited partial signals of isolation in Mediterranean, Balkan and perhaps Carpathian glacial refuges during Pleistocene [90]. Post-glacial recolonization determined a complex population mosaic, which has been further shaped by restrictions to gene flow due to local prey specialization, or by random drift due to recent anthropogenic fragmentation [91]. Consequently, wolf genetic diversity in Europe is geographically partitioned and populations are genetically identifiable [16]. Genetic divergence among wolf populations and between wolves and dogs provides the basis for a wealth of molecular markers that can be used in assignment and admixture analyses [88].

Admixed wolf genotypes may originate in consequence of intentional or accidental escapes of non-indigenous wolves from captivity, or by crossbreeding with dogs. In this study we did not detect consistent signals of admixture between Italian and other wolves. All the presumed hybrids clustered very close to - or partially overlapped with - the Italian wolves and showed no obvious connection to any other group. An exception was the strongly admixed sample n. 11, which was partially assigned to the WIT, WHR and WDCZ clusters. This sample was collected in the northern Apennines during 2011, showed the Italian wolf W14 mtDNA haplotype and a private YH32 Y-haplotype that was not found in any other wolf or dog group analyzed in this study (Table 4). Ancestry of this sample with dogs, non-Italian wolves or WDCZ cannot be excluded, although the haplotype YH32 was not found in the WDCZ samples. Most of the HYIT showed the Italian wolf haplotypes YH17 and YH26, but 2 individuals had the haplotype YH05, shared with DIT and DAP and that was also detected in WDCZ. The position of WDCZ in the DAPC plots indicates a higher proportion of dog genome. This is in agreement with the origin of the breed that was established in the 50ies by crossing 4 Carpathian wolf founders with German Shepherd dogs. Backcrossing with German Shepherds continued together with artificial selection. The standard of the current breed was approved by the Fédération Cynologique Internationale in 1989. The genotypes of WDCZ in the DAPC were not intermediate relative to their parental populations (DCZ and WCZ), probably in consequence of strong founder effect, persistent low effective population size and genetic drift. Moreover, captive wolfdogs experienced artificial selection designed to keep dog behaviour while preserving wolf-like phenotypic traits including coat colour, sensory abilities and endurance, with possible hitchhiking effects on linked neutral loci. Unofficial recurrent and more recent crossbreeding of wolf-like dogs with wolves by individual breeders may continue to generate hybrids with variable phenotypes and behaviours [92]. Hybrids may be aggressive, difficult to control and have chances to survive in nature crossbreeding with wolves. Genetic analyses of larger sample sizes are needed to identify local hybridization events, but, with the exception of the uncertain origin of sample n. 11 and haplotype YH05, the available evidence led to exclude that the WDCZ are a widespread source of hybridization with wolves in Italy, pointing out a main contribution of village and other dogs.

The *K* ^B^ melanistic deletion was detected only in DIT, DAP and HYIT. A different melanistic mutation at the *MC1R* gene is known to determine black coats specifically in German Shepherd dogs [75], [76] and explains also why WDCZ do not have the *K* ^B^ deletion. Wolf samples collected in Croatia and in the Carpathians were all wild-type grey and the *K* ^B^ deletion was not expected. We do not know the origin of the *K* ^B^ haplotype in the Italian wolves, if via hybridization with black dogs or by a spontaneous mutation at the *β-defensin CBD103* gene. The *K* ^B^ deletion was already present in ancient canids in Europe over 10 000 years ago, probably entered in North American wolf populations through ancient hybridization with dogs, and was also found in a melanistic pack of hybrid origin in Italy [28], [36], [93]. However these findings are still controversial and can not be generalized. Results of admixture analyses reported in our study are not unequivocal: 2 black-coated individuals showed strong signals of admixture at their multilocus STR genotypes and the concomitant presence of the melanistic *K* ^B^ deletion, while other 5 black-coated individuals did not show any signal of admixture. These animals, which could originate from past hybridization no longer detectable at their 39 STR genotypes, deserve additional investigations. The origin of the *K* ^B^ deletion in dog or wolf ancestors could be ascertained by sequencing the flanking haplotypes in Italian wolves and village dogs. Signals of past hybridizations may become detectable using genomic data and haplotype block reconstructions [3], [15], [16]. Melanistic phenotypes in wolves and dogs can be determined also by epistatic interactions among other and still undescribed mutations [94]. It is noteworthy that one Italian dark-coated backcross did not show the *K* ^B^ deletion (sample n. 9 in Table 4), suggesting that mutations at other structural or regulatory genes may add complexities to the expression of melanistic coat colour variations in wolves. A lack of samples or the absence of hybridization may explain why melanistic wolves were not found in other European countries (but see [29]).

We found that 50% of our putative hybrids showed unequivocal signals of Italian wolf *x* dog admixture at STRs. Seven of them also showed morphological anomalies, and 4 had the *K* ^B^ deletion or mtDNA CR1 and Y-STR haplotypes not found in the Italian wolves. Nine genotypes yielded weaker STR admixture signals, but showed dark coats, white nails, the *K* ^B^ deletion or variant mtDNA CR1 and Y-STR haplotypes. Hence, 24 (80%) of the putative hybrids showed combinations of variant phenotypic and genetic traits suggesting admixed origins. The remaining 6 samples were identified as presumptive hybrids only by Structure analyses with 12 STRs, which however might produce false positives. The putative hybrids were not randomly collected and the admixed individuals are not representative of the frequency of hybridization in the Italian wolf population. The frequency of hybridization should be estimated by extensive sampling through the entire wolf distribution range. A well planned country-wide program of wounded or found-dead wolf sampling would provide additional, but likely biased information, because the probability to encounter dead or wounded wolves is expected to vary in the heterogeneous landscapes used by wolves [95]. Moreover, carcasses of introgressed individuals can be confused with dogs and not collected [29]. Instead, exhaustive sampling collection throughout the wolf distribution range can be obtained by long-term non-invasive genetic monitoring programs [28].

Hybridization in wolves seems to be prevalently asymmetric, originating by female wolves mating with male dogs. The vast majority of admixed wolf genotypes described so far showed wolf mtDNA haplotypes [29], [31], [32], with a few exceptions [30], [52]). Mating of male wolves *x* female dogs, however, could occur because young males disperse frequently and are expected to explore and colonize new areas more rapidly than females [54], [96]. In this study, sample n. 1, an F1 identified by all the admixture analyses and confirmed by the allelic composition of all its STR loci, showed dog-like body shape and the mtDNA haplotype D15 that is shared only with Italian village and Apennine dogs, indicating a female dog parental. Moreover, we identified 3 backcrosses that shared the same mtDNA haplotype W16 so far detected only in Bulgarian wolves [40]. These samples were collected from carcasses found in 3 geographically distant areas of the northern Apennines during different years (2007, 2010 and 2011). Theoretically, they might originate from the same or a few related packs. However, their average Queller and Goodnight’s relatedness *r* = 0.076 was significantly lower (*P*<0.001, *t*-test) than average *r* estimated with the same panel of 12 STRs in 26 Italian wolf packs with known pedigrees (*r = *0.390±0.106 [28]), suggesting independent crossbreeding events. Thus, hybridization of wolves in Italy was not strictly patrilineal. Two other backcrosses had the Y-haplotype YH05, which was found in dogs sampled in Italy and in WDCZ. These samples were collected in 2 distant areas (central and southern Apennines) in 2009 and 2012. Probably they were not closely related (average *r* = − 0.128) and have originated from 2 independent hybridization events.

Simulations showed that *c*. 48 STRs with F_ST_ >0.10–0.15 are needed to significantly improve the reliability of backcross identifications [44]. VonHoldt et al. [38] demonstrated that even 100 highly diagnostic SNPs cannot efficiently discriminate second generation wolf *x* dog backcrosses. Thus, estimating the minimum number of markers to identify backcrosses is still an open issue. The outcomes of our admixture analyses computed using 39, 24 and 12 STRs were not as straightforward as expected. The estimate of admixed individuals did not increase using more loci, and a naïve assumption that larger panels of markers should lead to identify more admixed individuals was not fulfilled. The 24 most discriminating STRs were equally or more efficient than the full set of 39 STRs. Individual assignments were consistent for the 16 genotypes with the lowest *q*_i_ values in the Italian wolf cluster, which were also identified by GeneClass, independently on the assumptions embedded in the algorithms implemented in the different software. The assignments of the other genotypes were less consistent, and variable outcomes were obtained using 12 STRs. Some genotypes had disproportionally high *q*_i_ values (particularly running Structure with *popflag* = 0; e.g., n. 5 and 12) and could represent false negatives. Other samples showed disproportionally low *q*_i_ values (e.g., n. 25, 26, 27, 28, 29 and 30) and could be false positives. These results highlight 2 related issues that were often neglected in other studies:

1. HybridLab simulations showed how the power to correctly identify known (simulated) hybrids and backcrosses changes with the number of markers: the larger is the number of STRs, the higher is the threshold. Decreasing the number of markers decreases the average proportion of membership in reference clusters due to increasing background noise. Consequently, the width of CI values and the individual assignment uncertainty will increase. The risk of false admixed individuals is inversely proportional to the number of STRs, as indicated by BAPS simulations. The use of more markers allows to apply higher *q*_i_ thresholds, reducing uncertainty and the risk of false positives. Therefore, each study should plan adequate power analyses to identify the appropriate thresholds, whereas the adoption of threshold used in other studies might not guarantee optimal assignments.
2. The number of markers used in admixture analyses is not important *per se*, but the discriminating power of markers deeply affects the results of the assignments. The power of markers can be approximated by single-locus F_ST_ values between reference sample groups or assessed by other computational approaches [48], [72]. Selecting the most discriminating STRs will reduce the costs (both manpower and chemicals) of genetic assays, the rate of genotyping errors intrinsically associated to each additional marker, and the risk of false positives. The 24 most discriminating STRs selected in our study only partially overlapped the most discriminating STRs identified by Godinho et al. [29], indicating that the selection of loci should be performed based on local populations’ data sets. A similar approach was suggested by Hindrikson et al. [30], for mtDNA and Y haplotype identification.

Although genomic platforms promise extensive screening of thousands of SNPs, practical and financial constraints still limit their applications in conservation genetics. Genetic monitoring of carnivores is still based on the genotyping of limited numbers of STRs, often in DNAs extracted from non-invasively collected samples. In other cases, tissue samples are collected from found-dead animals, which often produce degraded DNA. Genotyping large numbers of STRs will probably continue to be problematic in practical conservation genetics, due to risks of false alleles and allelic dropout in molecular identifications of low-content DNA. For the same reasons, genotyping large numbers of SNPs is still unpractical in non-invasive genetics. Selecting the minimum number of informative autosomal STRs, plus informative mtDNA and Y-linked markers will remain the most viable strategy in the near future.

## Conclusions

The frequency of backcrosses or introgressed individuals (87.5%) between wolf and dog is far higher than the frequency of F1 and F2 hybrids (12.5%), suggesting that hybridization events already occurred in Italy some generations in the past. Probably this happened during the early phases of population re-expansion in Italy, when wolves moved from their historical core areas in the central-southern Apennines and colonized the northern Apennine mountains and lower hills [61]. Theoretical expectations [97] and empirical findings [29], [43] indicate that the risk of hybridization is higher in the periphery of wolf distributions in human-dominated landscapes, where wolf populations are less dense, free-ranging dogs are more abundant and early dispersing wolves have more probabilities to meet and mate with dogs. Expanding wolf populations will inevitably spread further into anthropogenically altered areas, where settlement density, infrastructure and the presence of agricultural activities will likely increase traffic casualties, illegal wolf killings. Consequently high pack turnover can contribute to further raise hybridization frequency. These findings suggest that: 1) expanding wolf populations may experience higher hybridization risks than stable populations; 2) the dynamics of hybridization and introgression will change through time, with a maximum expectancy of hybridization during the early phases of the colonization waves, followed by the subsequent spread of hybrids and the generation of backcrosses within wild populations. The spatial and temporal dynamics of hybridization and backcrossing should be conditioned by landscape features, anthropogenic factors, wolf and feral dog initial population density and colonization rates. These variables could be modelled using landscape genetic tools to reconstruct maps of hybridization risks, thus providing important resources for the monitoring and management of wolf populations in Europe.

## Supporting Information

Table S1 **Description of the genotyped autosomal (CFA) and Y-linked (CFAY) microsatellites (STR),** ***Amelogenin*** **and** ***β-defensin CBD103*** **(** ***K*** **-locus) genes, and the hypervariable part of the mtDNA control-region (mtDNA CR1).**
(DOC)

Table S2 **Values of the average proportions of membership of dogs (DIT, DAP and DCZ), wolves (WIT, WCZ, WHR), Czechoslovakian wolfdogs (WDCZ) and putative hybrids (HYIT) from Italy in** ***K*** **= 4 clusters computed with Structure (39 autosomal STRs,** ***admixture*** **and** ***I*** **models,** ***popflag*** **= 0).**
(DOC)

Table S3 **Admixture analyses in dogs (DIT, DAP and DCZ), wolves (WIT) and putative hybrids (HYIT) from Italy.** Values of the average proportions of membership of each sampled group in *K* = 4 clusters computed with Structure.
(DOC)

Text S1 **Description of laboratory methods with details on primers and PCR profiles for all the genotyped markers.**
(DOC)
