# Correction: Multilocus Detection of Wolf *x* Dog Hybridization in Italy, and Guidelines for Marker Selection

**DOI:** 10.1371/journal.pone.0091412

**Published:** 2014-03-25

**Authors:** The PLOS ONE Staff

In the Funding section, the number of an additional grant from the Czech University of Life Sciences Prague is incorrectly omitted from the Funding statement. The Funding statement should read: “This project was supported by grants from the Italian Ministry of Environment (MATTM), the Italian Institute for Environmental Protection and Research (ISPRA), the Czech University of Life Sciences Prague (IGA FTZ CZU 20135107 and 511120/1312/3108) and the International Visegrad Fund. The funders had no role in study design, data collection and analysis, decision to publish, or preparation of the manuscript.”

Figure 2 is incorrect. The authors have provided a corrected version here.

**Figure 2:**
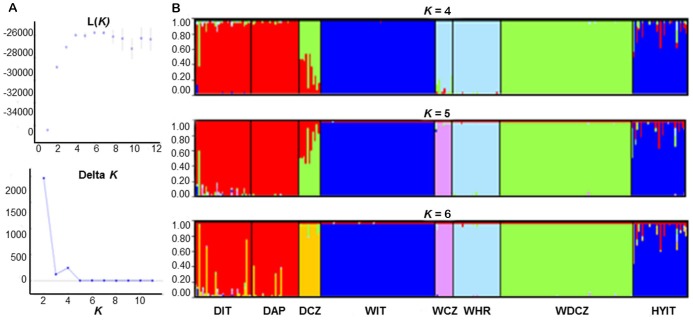
Structure analyses performed to infer the optimal partition of 8 sampled groups (A): DIT  =  village dogs in Italy; DAP  =  Apennine dogs; DCZ  =  German Shepherd; WIT  = wolves in Italy; WCZ  =  wolves in Czech and Slovak republics; WHR  =  wolves in Croatia; WDCZ  =  Czechoslovakian wolfdogs; HYIT  =  putative wolf *x* dog hybrids collected in Italy; (genotyped at 39 autosomal microsatellites). The posterior probability Ln(*K*) of the data and the statistics Δ*K* were used to identify the optimal *K*  =  4 (averages of two independent runs). Plots of individual assignment probability to each inferred cluster are shown (B) for optimal *K*  =  4, 5 and 6. Structure was run assuming *K* from 1 to 12, with 400,000 MCMC and discarding the first 40,000 burn-ins, using the “*admixture*” and independent allele frequency “*I*” models, and no prior information (option “*usepopinfo*” not activated).

## References

[1] Randi E, Hulva P, Fabbri E, Galaverni M, Galov A, et al (2014) Multilocus Detection of Wolf x Dog Hybridization in Italy, and Guidelines for Marker Selection. PLoS ONE 9(1): e86409 doi:10.1371/journal.pone.0086409 24466077 10.1371/journal.pone.0086409 PMC3899229

