# Supplementary material for: Multilocus Detection of Wolf x Dog Hybridization in Italy, and Guidelines for Marker Selection
Source: PLoS One. 2014 Jan 22;9(1):e86409. doi: 10.1371/journal.pone.0086409 (PMC3899229; doi:10.1371/journal.pone.0086409)
Supplement: Table S1 — Description of the genotyped autosomal (CFA) and Y-linked (CFAY) microsatellites (STR), Amelogenin and β-defensin CBD103 ( K -locus) genes, and the hypervariable part of the mtDNA control-region (mtDNA CR1). (DOC) [file pone.0086409.s001.doc]

Table S1. Description of the genotyped autosomal (CFA) and Y-linked (CFAY) microsatellites (STR), *Amelogenin* and *β-defensin CBD103* (*K*-locus) genes, and the hypervariable part of the mtDNA control-region (mtDNA CR1).

| **Locus** | **Chromosome** | **STR**  **repeat size** | **Allele sizes**  **(bp)** | **Dye label** | **Multiplex** | **Reference** | **Genomic**  **context**a | **Gene location /**  **Nearest gene**a | **Source**a |
| --- | --- | --- | --- | --- | --- | --- | --- | --- | --- |
| AHTk211 | CFA26 | Dinucleotide | 79-101 | FAM | MF | [1] | Inter-genic | *LHX5* | HO |
| CXX279 | CFA22 | Dinucleotide | 109-133 | FAM | MF | [2] | Inter-genic | *CLYBL* | HO |
| REN169O18 | CFA29 | Dinucleotide | 150-170 | FAM | MF | [3] | Inter-genic | *CAP1* | HO |
| INU055 | CFA10 | Dinucleotide | 190-216 | FAM | MF | Finnzymes | Inter-genic | *ETAA1* | HO |
| REN54P11 | CFA18 | Dinucleotide | 222-244 | FAM | MF | [3] | Intronic | *MAGI2* | HO |
| AHT137 | CFA11 | Dinucleotide | 126-156 | HEX | MF | [4] | Inter-genic | *UBTD2* | HO |
| REN169D01 | CFA14 | Dinucleotide | 199-221 | HEX | MF | [3] | Inter-genic | *ABCB5* | HO |
| AHTh260 | CFA16 | Dinucleotide | 230-254 | HEX | MF | [5] | NA | NA | NA |
| AHTk253 | CFA23 | Dinucleotide | 277-297 | HEX | MF | [1] | Inter-genic | *RPS2P32* | HO |
| INU005 | CFA33 | Dinucleotide | 102-136 | NED | MF | Finnzymes | Intronic | *PARP9* | HO |
| INU030 | CFA12 | Dinucleotide | 139-157 | NED | MF | Finnzymes | Intronic | *GRIK2* | HO |
| FH2848 | CFA2 | Dinucleotide | 222-244 | NED | MF | [5] | Inter-genic | *LINC00710* | HO |
| REN162C04 | CFA7 | Dinucleotide | 192-212 | PET | MF | [3] | Intronic | *DNM3* | HO |
| AHTh171 | CFA6 | Dinucleotide | 215-239 | PET | MF | [5] | Intronic | *SRM3* | HO |
| REN247M23 | CFA15 | Dinucleotide | 258-282 | PET | MF | [3] | Inter-genic | *LIN7A* | HO |
| FH2004 | CFA11 | Tetranucleotide | 104-202 | PET | M1 | [6] | Intronic | *PTPRD* | RS, HO |
| FH2088 | CFA15 | Dinucleotide | 91-139 | FAM | M1 | [6] | Inter-genic | *FHDC1* | HO |
| FH2096 | CFA11 | Tetranucleotide | 86-110 | HEX | M1 | [6] | Inter-genic | *DDX43* | HO |
| FH2137 | CFA3 | Dinucleotide | 140-192 | HEX | M1 | [6] | Inter-genic | *CHD1* | HO |
| CPH2 | CFA32 | Dinucleotide | 88-106 | NED | M1 | [7] | Intronic | *SCD5* | RS, HO |
| CPH8 | CFA13 | Dinucleotide | 191-219 | FAM | M1 | [7] | Inter-genic | *LOC1720* | HO |
| FH2079 | CFA24 | Tetranucleotide | 246-282 | FAM | M2 | [6] | Inter-genic | *PTNP1* | HO |
| CPH4 | CFA15 | Dinucleotide | 130-155 | NED | M2 | [7] | Intronic | *ANKS1B* | HO |
| CPH5 | CFA15 | Dinucleotide | 102-124 | HEX | M2 | [7] | Inter-genic | *ATAD2B* | HO |
| CPH12 | CFA8 | Dinucleotide | 188-214 | FAM | M2 | [7] | Intronic | *NPAS3* | RG, HO |
| C09.250 | CFA9 | Dinucleotide | 121-145 | PET | M2 | [2] | Intronic | *NXN* | HO |
| C20.253 | CFA20 | Dinucleotide | 90-120 | NED | M2 | [2] | Intronic | *FHIT* | RS, HO |
| AHT132 | CFA2 | Dinucleotide | 160-172 | PET | M3 | N. Holmes | Inter-genic | *CALML3* | HO |
| C27.442 | CFA27 | Dinucleotide | 158-172 | HEX | M3 | [2] | Intronic | *SLC11A2* | HO |
| FH2010 | CFA24 | Tetranucleotide | 216-240 | NED | M3 | [6] | Intronic | *PLK1S1* | RG, HO |
| PEZ1 | CFA7 | Tetranucleotide | 99-131 | HEX | M3 | [8] | NA | NA | NA |
| PEZ5 | CFA12 | Tetranucleotide | 95-119 | PET | M3 | [8] | Inter-genic | *FBXL4* | HO |
| AHT103 | CFA4 | Dinucleotide | 71-89 | HEX | M4 | [4] | Inter-genic | *RANBP3L* | HO |
| AHT111 | CFA2 | Dinucleotide | 72-92 | NED | M4 | [4] | Intronic | *IL22RA1* | HO |
| FH2001 | CFA23 | Tetranucleotide | 123-155 | PET | M4 | [6] | Inter-genic | *ARHGEF26* | HO |
| C09.173 | CFA9 | Dinucleotide | 100-118 | FAM | M4 | [2] | Intronic/Exonic | *ABCA5* | RS, HO |
| C13.758 | CFA13 | Dinucleotide | 220-244 | NED | M4 | [9] | Inter-genic | *FER1L6* | HO |
| CPH9 | CFA28 | Dinucleotide | 139-151 | HEX | M4 | [7] | Inter-genic | *ZFHX4-AS1* | HO |
| CPH14 | CFA5 | Dinucleotide | 185-205 | PET | M4 | [7] | NA | NA | NA |
|  |  |  |  |  |  |  |  |  |  |
| MSY34A | CFAY | Dinucleotide | 160-190 | NED | M5 | [10] | NA | NA | NA |
| MSY41A | CFAY | Dinucleotide | 90-150 | HEX | M5 | [10] | NA | NA | NA |
| MSY34B | CFAY | Dinucleotide | 167-177 | HEX | M5 | [10] | NA | NA | NA |
| MSY41B | CFAY | Dinucleotide | 109-137 | NED | M5 | [10] | NA | NA | NA |
|  |  |  |  |  |  |  |  |  |  |
| *Amelogenin* | CFAX | - | 174-218 | NED | MF | Finnzymes | Intronic | *Amelx* | RS,HO |
| *K*-locus | CFA16 | Codon deletion | 147-151 | HEX | M2 | [11, 12] | Esonic/Intronic | *CBD103* | RG,RS,HO |
| mtDNA CR1 | mtDNA | - | 350 | - | - | [13] | Control-region | *Cytochrome b* | RS |

^a^ Genomic context, gene location, source. Primer locations along the reference dog genome (CanFam3.1 assembly) have been identified either via the UniSTS database at NCBI (<http://www.ncbi.nlm.nih.gov/genome/sts/>), or by in-silico PCR via the USCS genome browser (<http://genome.ucsc.edu/>). The UCSC genome browser was also used to identify the genomic context of each STR, which were classified as inter-genic, intronic or exonic, in relation to the annotated dog genes available from RefGene (RG), RefSeq (RS), or to the presence of human orthologous transcripts (HO). NA = locus not mapped in the reference dog chromosomes (CanFam3.1 assembly).

**References**

1. Thomas R, Holmes N, Fischer P, Dickens H, Breen M, et al. (1997) Eight canine microsatellites. Animal Genetics 28: 153-154.

2. Ostrander EA, Sprague GF, Rine J (1993) Identification and characterization of dinucleotide repeat (CA) markers for genetic mapping in dog. Genomics 16: 207-213.

3. Guyon R, Lorentzen TD, Hitte C, Kim L, Cadieu E, et al. (2003) A 1-Mb resolution radiation hybrid map of the canine genome. Proceedings of the National Academy of Sciences 100: 5296-5301.

4. Holmes N, Humphreys S, Binns M, Holliman A, Curtis R, et al. (1993) Isolation and characterization of microsatellites from the canine genome. Animal Genetics 24: 289-292.

5. Breen M, Jouquand S, Renier C, Mellersh CS, Hitte C, et al. (2001) Chromosome-specific single-locus FISH probes allow anchorage of an 1800-marker integrated radiation-hybrid/linkage map of the domestic dog genome to all chromosomes. Genome Research 11: 1784-1795.

6. Francisco L, Langsten A, Mellersh C, Neal C, Ostrander E (1996) A class of highly polymorphic tetranucleotide repeats for canine genetic mapping. Mammalian Genome 7: 359-362.

7. Fredholm M, Winterø A (1995) Variation of short tandem repeats within and between species belonging to the Canidae family. Mammalian Genome 6: 11-18.

8. Neff MW, Broman KW, Mellersh CS, Ray K, Acland GM, et al. (1999) A second-generation genetic linkage map of the domestic dog, *Canis familiaris*. Genetics 151: 803-820.

9. Mellersh CS, Langston AA, Acland GM, Fleming MA, Ray K, et al. (1997) A linkage map of the canine genome. Genomics 46: 326-336.

10. Sundqvist AK, Ellegren H, Olivier M, Vila C (2001) Y chromosome haplotyping in Scandinavian wolves (*Canis lupus*) based on microsatellite markers. Molecular Ecology 10: 1959-1966.

11. Candille SI, Kaelin CB. Cattanach BM, Yu B, Thompson DA, Nix MA, Kerns J, Schmutz SM, Millhauser GL, and Barsh GS (2007) A *β-defensin* mutation causes black coat color in domestic dogs. Science 318: 1418-1423.

12. Caniglia R, Fabbri E, Greco C, Galaverni M, Manghi L, Boitani L, Sforzi A, Randi E (2013) Black coats in an admixed wolf × dog pack: is melanism an indicator of hybridization in wolves? European Journal of Wildlife Research 59: 543-555.

13. Randi E, Lucchini V, Christensen MF, Mucci N, Funk SM, et al. (2000) Mitochondrial DNA variability in Italian and East European wolves: Detecting the consequences of small population size and hybridization. Conservation Biology 14: 464-473.
