# Supplementary material for: Multilocus Detection of Wolf x Dog Hybridization in Italy, and Guidelines for Marker Selection
Source: PLoS One. 2014 Jan 22;9(1):e86409. doi: 10.1371/journal.pone.0086409 (PMC3899229; doi:10.1371/journal.pone.0086409)
Supplement: Table S2 — Values of the average proportions of membership of dogs (DIT, DAP and DCZ), wolves (WIT, WCZ, WHR), Czechoslovakian wolfdogs (WDCZ) and putative hybrids (HYIT) from Italy in K = 4 clusters computed with Structure (39 autosomal STRs, admixture and I models, popflag = 0). (DOC) [file pone.0086409.s002.doc]

Table S2. Values of the average proportions of membership of dogs (DIT, DAP and DCZ), wolves (WIT, WCZ, WHR), Czechoslovakian wolfdogs (WDCZ) and putative hybrids (HYIT) from Italy in *K* = 4 clusters computed with Structure (39 autosomal STRs, *admixture* and *I* models, *popflag* = 0).

| **Group** | **Cluster 1** | **Cluster 2** | **Cluster 3** | **Cluster 4** |
| --- | --- | --- | --- | --- |
| DIT | 0.940 | 0.010 | 0.017 | 0.033 |
| DAP | 0.990 | 0.001 | 0.002 | 0.007 |
| DCZ | 0.337 | 0.001 | 0.001 | 0.661 |
| WIT | 0.001 | 0.997 | 0.001 | 0.001 |
| WCZ | 0.016 | 0.004 | 0.914 | 0.066 |
| WHR | 0.003 | 0.004 | 0.989 | 0.003 |
| WDCZ | 0.002 | 0.001 | 0.002 | 0.994 |
| HYIT | 0.057 | 0.906 | 0.007 | 0.029 |
