# Supplementary material for: Multilocus Detection of Wolf x Dog Hybridization in Italy, and Guidelines for Marker Selection
Source: PLoS One. 2014 Jan 22;9(1):e86409. doi: 10.1371/journal.pone.0086409 (PMC3899229; doi:10.1371/journal.pone.0086409)
Supplement: Table S3 — Admixture analyses in dogs (DIT, DAP and DCZ), wolves (WIT) and putative hybrids (HYIT) from Italy. Values of the average proportions of membership of each sampled group in K = 4 clusters computed with Structure. (DOC) [file pone.0086409.s003.doc]

Table S3. Admixture analyses in dogs (DIT, DAP and DCZ), wolves (WIT) and putative hybrids (HYIT) from Italy. Values of the average proportions of membership of each sampled group in *K* = 4 clusters computed with Structure.

| **Structure *Popinfo* inactive^a^** | | | | | **Structure *Popinfo* active^b^** | | | |
| --- | --- | --- | --- | --- | --- | --- | --- | --- |
| **Group** | **Cluster 1** | **Cluster 2** | **Cluster 3** | **Cluster 4** | **Cluster 1** | **Cluster 2** | **Cluster 3** | **Cluster 4** |
| DIT | 0.632 | 0.201 | 0.161 | 0.006 | 0.863 | 0.044 | 0.093 | 0.000 |
| DAP | 0.009 | 0.912 | 0.077 | 0.002 | 0.002 | 0.970 | 0.028 | 0.000 |
| DCZ | 0.002 | 0.005 | 0.992 | 0.001 | 0.000 | 0.000 | 1.000 | 0.000 |
| WIT | 0.001 | 0.001 | 0.001 | 0.997 | 0.000 | 0.000 | 0.000 | 1.000 |
| HYIT | 0.026 | 0.030 | 0.041 | 0.903 | 0.036 | 0.031 | 0.038 | 0.891 |

^a^ Structure analyses performed using 39 STRs, *admixture* and *I* models, *popflag* = 0 (*Popinfo* inactive).

^b^ Structure analyses performed using 39 STRs, *admixture* and *I* models, *popflag* = 1 (*Popinfo* active).
