# Supplementary material for: Multilocus Detection of Wolf x Dog Hybridization in Italy, and Guidelines for Marker Selection
Source: PLoS One. 2014 Jan 22;9(1):e86409. doi: 10.1371/journal.pone.0086409 (PMC3899229; doi:10.1371/journal.pone.0086409)
Supplement: Text S1 — Description of laboratory methods with details on primers and PCR profiles for all the genotyped markers. (DOC) [file pone.0086409.s004.doc]

**Text S1. Description of laboratory methods with details on primers and PCR profiles for all the genotyped markers**

The microsatellites in the Finnzymes Canine multiplex kit (Finnzymes, Thermo Scientific Canine Genotypes^TM^) and the *Amelogenin* locus were amplified in a single multiplex PCR reaction (MF) using an Applied Biosystems Thermal Cycler (ABI GeneAmp® PCR System 9700) with the following thermal profile: 98°C/3 min, 98°C/15 sec, 60°C/90 sec, 72°C/30 sec (30-40 cycles), followed by a final extension step at 72°C for 5 min. The amplifications were carried out in a 20 μl total PCR volume, including 2 μl of DNA solution from saliva samples, or 1 μl of DNA solution from muscle and blood samples, corresponding to *c*. 20 – 40 ng of DNA, 10 μl of Finnzymes Canine Genotypes™ Panel 1.1 Master Mix (which included an optimized buffer containing MgCl2, deoxynucleoside triphosphates (dNTP) and Phusion™ Hot Start DNA Polymerase with an activity of 0.05 U/μl), and 10 μl of Finnzymes Canine Genotypes™ Panel 1.1 Primer Mix (including forward and reverse primers for the 19 markers).

The autosomal and Y-linked STR loci were amplified in other 5 multiplexed primer mixes (M1, M2, M3, M4, M5) using the Qiagen Multiplex PCR Kit (Qiagen Inc, Hilden, Germany), an ABI GeneAmp® PCR System 9700, and the following thermal profile: 94°C/15 min, 94°C/30 sec, 57°C/90 sec, 72°C/60 sec (30 cycles), followed by a final extension step at 72°C for 5 min. Amplifications were carried out in 10 μl total volume, including 2 μl of DNA solution from saliva samples, or 1 μl of DNA solution from muscle and blood samples, 5 μl Qiagen Multiplex PCR mix, 1 μl Qiagen Q solution, 0.4 μM deoxynucleotide triphosphates (dNTP), from 0.1 μl to 0.4 μl of 10 μM primer mix (forward and reverse) and RNase-free water up to the final volume.

The 3-bp deletion (named *K^B^* or *CBD103^ΔG23^*) at the *β-defensin CBD103* gene (the *K*-locus) was genotyped following Caniglia et al. [1], in 10 μl PCR volumes including 1 μl or 2 μl of DNA solution and 0.3 pmol of the primers CBD103_ΔG23F (TCCGGCACGTTCTGTTTT, 6-FAM) and CBD103_ΔG23R (TTCGGCCAGTGGAAGAAC) that amplify a fragment of 190/193 bp. PCR conditions were: 94°C/2 min, 94°C/15 sec, 55°C/15 sec, 72°C/30 sec (40 cycles), plus one final cycle at 72°C for10 min.

The mtDNA control-region was amplified in 10 μl PCR volumes, including 1 μl or 2 μl of DNA solution, 0.3 pmol of the primers L-Pro and H350 (Randi et al. [2]), using the following thermal profile: 94°C/2 min, 94°C/15 sec, 55°C/15 sec, 72°C/30 sec (40 cycles), followed by a final extension at 72°C for 5 min. PCR products were purified using the exonuclease/shrimp alkaline phosphatase procedure (Exo-Sap; Amersham) and sequenced in both directions using the ABI Big Dye Terminator kit with the following steps: 96°C/10 sec, 55°C/5 sec, 60°C/4 min of final extension (25 cycles).

**References**

1. Caniglia R, Fabbri E, Greco C, Galaverni M, Manghi L, Boitani L, Sforzi A, Randi E (2013) Black coats in an admixed wolf *×* dog pack: is melanism an indicator of hybridization in wolves? European Journal of Wildlife Research 59: 543-555.

2. Randi E, Lucchini V, Christensen MF, Mucci N, Funk SM, et al. (2000) Mitochondrial DNA variability in Italian and East European wolves: Detecting the consequences of small population size and hybridization. Conservation Biology 14: 464-473.
